# Are morbidity and mortality estimates from randomized controlled trials externally valid? A comparison of outcomes among infants enrolled into an RCT or a cohort study in Botswana

**DOI:** 10.1186/s12874-021-01343-5

**Published:** 2021-10-17

**Authors:** Neil Thivalapill, Shahin Lockman, Kathleen Powis, Rebecca Zash, Jean Leidner, Gbolahan Ajibola, Mompati Mmalane, Joseph Makhema, Roger L. Shapiro

**Affiliations:** 1 Department of Immunology and Infectious Diseases, Harvard T.H. Chan School of Public Health, 651 Huntington Ave FXB 401, Boston, MA 02115 USA; 2 Botswana Harvard AIDS Institute Partnership, Gaborone, Botswana; 3 Harvard Medical School, Boston, MA USA; 4 Brigham and Women’s Hospital, Boston, MA USA; 5 Massachusetts General Hospital, Boston, MA USA; 6 Beth Israel Deaconess Medical Center, Boston, MA USA; 7 Goodtables Data Consulting, Norman, OK USA

**Keywords:** External validity; Clinical trials; Implementation science; HIV-Exposed Uninfected (HEU); Pragmatic trials; Treatment effects

## Abstract

**Background:**

The external validity of the randomized controlled trial (RCT) refers to the extent to which the results of the RCT apply to the relevant, non-trial population and is impacted by its eligibility criteria, its organization, and its delivery of the intervention. Here, we compared the outcomes of mortality and hospitalization between an RCT and a cohort study that concurrently enrolled HIV-exposed uninfected (HEU) newborns in Botswana.

**Methods:**

The Mpepu Study (the RCT) was a clinical trial which determined that co-trimoxazole (CTX) provided no survival benefit for HEUs, allowing both arms of the RCT to be used. The Maikaelelo study (the cohort study) was a prospective observational study that enrolled HEU newborns with telephone follow-up and no in-person visits. Rates of death and hospitalization in the pooled population, were modeled using cox-proportional hazards models for time to death or time to first hospitalization, with study setting (RCT vs. cohort study) as an independent variable. The causal effect of study setting on morbidity and mortality was obtained through a treatment effects approach.

**Results:**

In total, 4,010 infants were included; 1,306 were enrolled into the cohort study and 2,704 were enrolled into the RCT. No significant differences in mortality were observed between the two study settings (HR: 1.28, 95% CI: 0.76, 2.13), but RCT participants had a lower risk of hospitalization (HR: 0.72, 95% CI: 0.58, 0.89) that decreased with age. However, RCT participants had a higher risk of hospitalization within the first six months of life. The causal risk difference in hospitalizations attributable to the RCT setting was -0.03 (95% CI: -0.05, -0.01).

**Conclusions:**

Children in an RCT with rigorous application of national standard of care guidelines experienced a significantly lower risk of hospitalization than children participating in a cohort study that did not alter clinical care. Future research is needed to further investigate outcome disparities when real-world results fail to mirror those achieved in a clinical trial.

Trial registration

The Mpepu Trial was funded by the U.S. National Institutes of Health (No. NCT01229761) and the Maikaelelo Study was funded primarily by the U.S. Centers for Disease Control and Prevention (32AI007433-21).

## Background

Randomized controlled trials (RCTs) are often considered the highest grade of evidence in clinical research because the randomization process aims to balance both known and unknown confounders [1]. In doing so, these trials seek to measure the true causal effect of an intervention on a given outcome [2]. Historically, much of the literature discusses “internal” aspects of trial execution that may introduce bias to the estimation of causal effect [3]. What is less discussed, however, is the external validity of clinical trials – that is, the extent to which the results of a clinical trial can be applied to a relevant population of interest [4, 5].

There are various dimensions to the conduct of clinical trials that improve the validity of their results in the real-world setting. The PRECIS-2 tool considers nine of these domains, each of which is scored on whether the trial answers questions about pragmatism under real-world conditions or efficacy under ideal conditions [6]. These domains include the eligibility criteria, the recruitment of study subjects, the setting in which the study was conducted, the organization of the study, the delivery of the intervention and the adherence to it, the follow-up methods used, the primary outcome measured, and the methods of the primary analysis.

The concerns that the idealistic conduct of a trial deviates from routine operations of care in the real-world have led to a movement toward both pragmatic clinical trials and implementation science [7]. This is to say that there are two mechanisms through which the standard of care in an RCT could be different from the real-world setting. The first is one in which the care provided by an RCT is elevated compared to what is offered in the real-world setting because the design of the study itself includes services that have been added to routine care. The alternative mechanism is often found in resource-limited settings, where the RCT provides what is intended to be routine care, which the real-world setting is unable to provide because of both health-systems and patient-level barriers. The two mechanisms require two different approaches to ensure harmony between RCT results and real-world results. In the first scenario, pragmatic trials are necessary to limit the disparity between what is offered and accessed in routine care and what is offered to RCT participants. In the second, an implementation science approach is necessary in order to ensure that the ideal outcomes reported in trials conducted with routine care are achieved in real-world settings.

Assessing external validity is difficult to do empirically for a number of reasons that are both methodological and practical. First, it is possible that there exist other domains apart from those proposed by the PRECIS-2 tool that could explain a lack of external validity between an RCT and a relevant, non-study population. Second, given the multi-faceted nature of external validity, no one measure could summarize all the relevant considerations. The literature on this subject has therefore often focused on assessing one or two of these dimensions at a time, most notably the representativeness of the trial population to the larger population about which the trial sought to make inferences [8]. More recent work has focused on attempting to reconcile effect estimates of interventions from RCTs to their effect estimates in relevant populations using real-world evidence platforms [9]. However, little empirical work has been done to assess how the domains of eligibility (who is selected to participate in the trial), setting (where the trial is being done), organization (the expertise and resources needed to deliver the intervention), and delivery (how the intervention is delivered) affect the external validity of an RCT. In this paper, we consider how these domains of the Mpepu Trial (henceforth “the RCT”) affected the rate of morbidity (hospitalization) and mortality (death) among HIV-Exposed Uninfected (HEU) infants compared to the observational Maikaelelo Study (henceforth “the cohort study”), which concurrently enrolled HEU infants.

While details of the design of both the RCT and the cohort study are listed below, it is important to note that the RCT was examining the mortality and morbidity benefit of co-trimoxazole (CTX) administered to HEU infants and determined that CTX provided no benefit. The cohort study was a prospective observational cohort study that followed morbidity and mortality outcomes of HEU infants. By leveraging the overlapping outcomes data from these two concurrent studies in Botswana, we captured the extent to which enrollment into the clinical trial setting affected both mortality and morbidity compared to a cohort study that represented routinely accessed care in Botswana. While the goal of this work is to determine whether the rates of morbidity and mortality obtained in the RCT setting were valid in the non-RCT context, we cannot comment on whether the effect estimate of CTX was valid given that the cohort study did not assess the efficacy of this intervention. Nevertheless, we believe that this work provides a new dimension to the puzzle of external validity, one that offers a quantitative measure of how standard of care and setting differences affected the external validity of morbidity and mortality rates between an RCT and a cohort study.

## Methods

The data for this analysis originates from two studies in Botswana: the Mpepu Trial (Clinical Trial No. NCT01229761**),** a randomized clinical trial, and the Maikaelelo Study, a prospective observational cohort study that captured data using telephone follow-up. Below, we briefly describe the designs of each study and in Additional File 1, we describe the relevant considerations of each study and its execution.

### The Mpepu Trial (RCT)

The Mpepu Trial was a double-blind, randomized, placebo-controlled trial in which 2,848 HEU infants enrolled within the first 34 days of life between June 2011 and April 2015 and randomized to receive CTX or placebo [10]. Women with documented HIV-1-infection were recruited from public antenatal clinics or maternity wards in southern Botswana between the 26^th^ week of pregnancy up to 34 days postpartum. The study was conducted in an area of Botswana without malaria transmission. Recruitment took place in Gaborone (urban setting), Molepolole (large village), and Lobatse (town). Mothers who elected to breastfeeding their infants gave consent to be randomly assigned to breastfeeding for 6 months (the recommended duration in Botswana) or 12 months (the duration recommended by WHO). Breastfed children were allocated by factorial randomization to CTX vs. Placebo and to 6 vs. 12 months of breastfeeding. The trial was stopped for futility as the data and safety monitoring board concluded a low likelihood of benefit with CTX.

### The Maikaelelo Study (Cohort Study)

The Maikaelelo Study was an observational cohort study that enrolled mother-infant pairs from five public hospital maternity wards in Botswana, including Francistown (urban setting), Mochudi (large village), Ramotswa (village), Maun (town), and Kanye (large village). Between January 2012 and March 2013, the Maikaelelo study enrolled 1,499 HIV-infected and 1,501 HIV-negative mothers and their 3,033 infants, of which 1,515 were HEU [11]. In the primary analysis of Maikaelelo, HIV-exposed children with unknown infection were considered HIV-uninfected.

A combined dataset of children enrolled in the Mpepu Trial and the Maikaelelo Study was created.

### Inclusion criteria

Infants were included if they were HEU, as determined by the definitions of the respective study. To be conservative in the approach, infants whose HIV status was unknown by the end of the study period were excluded.

### Exclusion criteria

Infants were excluded from the analysis if they died or were hospitalized prior to enrollment. Infants were also excluded from the analysis if maternal HIV treatment during pregnancy was missing. Infants from the Mpepu Study who were not randomized (and therefore did not enroll into the trial) were also excluded. Infants who died or were hospitalized within 30 days of birth were excluded from the analysis to ensure comparability between trials given their different enrollment strategies. Finally, events among infants in Maikaelelo that occurred after 547 days or 18 months (the length of follow-up in Mpepu) were excluded from the analysis.

### Clinical site

Given the non-overlapping clinical sites in Botswana from which these children were enrolled, an indicator variable was used to adjust for sites in large urban settings (Francistown and Gaborone) compared to sites in more rural or peri-urban settings (Maun, Ramotswa, Mochudi, Kanye, Lobatse, and Molepolole).

### Socioeconomic status

Since both studies collected comparable socioeconomic data, a socioeconomic score was created ranging from 0 to 7, with 0 corresponding to lower socioeconomic status and 7 corresponding to a higher socioeconomic status. This score was a summation of the scores of maternal education (from none/primary to secondary to university), household access to electricity (from no access to access), source of water (from not piped into the home to piped into the home), and housing structure (from no stable housing, to informal housing, to mixed formal/informal, and to formal housing).

### Outcomes

Outcomes of interest were death and hospitalization as binary outcomes, as well as time to death and time to first hospitalization.

### Covariates

Covariates included in all analyses were study setting (RCT vs. cohort study), sex, categorized site (Francistown/Gaborone vs. Other), socioeconomic status, breastfeeding strategy (ever breastfed vs. exclusively formula fed), maternal HIV treatment during pregnancy (none vs. zidovudine (ZDV) only vs. three-drug regimen), and low birthweight status (< 2500 g).

### Survival analysis

To understand the relationship between study setting (RCT vs. cohort study), time, and either of the outcomes of death or hospitalization, two Cox proportional hazards model were fit modeling either time to death or time to first hospitalization from 1–18 months, adjusted for the aforementioned covariates including study setting as an independent variable. Where the proportional hazards assumption was thought to be violated, an interaction term between the study setting variable and the analysis time was generated. An interaction term between the natural logarithm of analysis time and study setting was modeled using restricted cubic spline transformations to visually assess the changes in the hazard ratio over time.

### Causal effect estimation

The causal effect of study setting was obtained through an inverse probability-weighted estimator, and inferences were made with 100 replications of the bootstrapped standard errors. The results of this approach report two coefficients: the potential-outcome mean (POM) which is the risk of the outcome had all children been enrolled into the cohort study and the average treatment effect (ATE) which is the average difference in risk between the potential risk had all children been enrolled into the cohort study and the potential risk had all children been enrolled into the RCT. The treatment effects estimator attempts to make the treatment variable (study setting (RCT vs. cohort study)) and outcome variable (morbidity or mortality) independent after conditioning on the covariates of type of site (urban vs. non-urban), socioeconomic status, breastfeeding status, ARV strategy during pregnancy, and low-birthweight [12]. The function was implemented using the *teffects ipw* command in Stata.

### Sensitivity analyses

Sensitivity analyses were also conducted in which infants who died or were hospitalized within 30 days of enrollment into either study were excluded to determine whether these inferences held even after excluding the earlier effects of study setting.

## Results

### Baseline demographics

Table 1 describes the characteristics of the infants who were HEU presented by study setting. In total, 4,010 infants were included in the analysis, 1,306 (32.57%) from the cohort study and 2,704 (67.43%) from the RCT. Between 30 and 547 days of follow-up, death occurred in 23 (1.76%) infants in the cohort study and 53 (1.96%) infants in the RCT. Hospitalization occurred in 157 (12.02%) in the cohort study and 214 (7.91%) in the RCT.

**Table 1:** Baseline Demographic and Clinical Characteristics of the Mpepu (RCT) and Maikaelelo (Cohort) HEU Infants Enrolled

	**Maikaelelo (Cohort Study)**	**Mpepu** **(RCT)**	**p-value**
	N = 1,306	N = 2,704	
**Site Name**			< 0.001
Francistown	612 (46.86%)	0 (0.00%)	
Gaborone	0 (0.00%)	1,600 (59.17%)	
Kanye	148 (11.33%)	0 (0.00%)	
Lobatse	0 (0.00%)	201 (7.43%)	
Maun	343 (26.26%)	0 (0.00%)	
Mochudi	119 (9.11%)	0 (0.00%)	
Molepolole	0 (0.00%)	903 (33.39%)	
Ramotswa	84 (6.43%)	0 (0.00%)	
**Site Categorized**			< 0.001
Other	694 (53.14%)	1,104 (40.83%)	
Francistown/Gaborone	612 (46.86%)	1,600 (59.17%)	
**Gender**			0.79
Female	671 (51.38%)	1,377 (50.92%)	
Male	635 (48.62%)	1,327 (49.08%)	
**Breastfeeding Strategy**			< 0.001
Breastfed	184 (14.09%)	615 (22.74%)	
Exclusively FF	1,122 (85.91%)	2,089 (77.26%)	
**Breastfeeding Days**	245.07 (76.77)	172.03 (103.38)	< 0.001
**Birthweight**	2.95 (0.46)	2.93 (0.49)	0.24
**Low Birthweight**			0.22
< 2.5 kg	199 (15.24%)	453 (16.75%)	
Normal	1,107 (84.76%)	2,251 (83.25%)	
**ARV Regimen**			< 0.001
None	81 (6.20%)	23 (0.85%)	
ZDV Only	349 (26.72%)	350 (12.94%)	
3-Drug ART	876 (67.08%)	2,331 (86.21%)	
**Socioeconomic Status Score**	4.26 (1.28)	4.39 (1.20)	0.003
**Maternal Baseline CD4**	448.08 (232.51)	540.62 (260.32)	< 0.001
**Maternal Baseline VL**			< 0.001
< 400 copies/mm^3^	62 (4.75%)	1,047 (38.72%)	
> = 400 copies/mm^3^	154 (11.79%)	381 (14.09%)	
Missing	1,090 (83.46%)	1,276 (47.19%)	

The RCT enrolled more infants from large urban settings compared to the cohort study (59.17% vs. 46.86%, p < 0.001) and more mothers in the RCT chose to breastfeed their infants (22.74% vs. 14.09%, p < 0.001). Mothers in the RCT were more likely to be receiving 3-drug ART than no ARV strategy or ZDV only compared to mothers in the cohort study (86.21% vs. 67.08%, p < 0.001). On average, mothers in the RCT had a higher socioeconomic status (4.39 vs. 4.26, p = 0.003) and a higher baseline CD4 count (540.62 cells/mL^3^ vs. 448.08 cells/mL^3^, p < 0.001). No significant differences were observed between the two study settings with regards to sex (p = 0.79), mean birthweight (p = 0.24) or low birthweight status (p = 0.22) of the children. After applying the exclusion criteria mentioned earlier, no infants used in the analysis had missing outcomes.

## Survival analysis

### Mortality

In the cox proportional hazards model fitted for time to infant death (Table 2), and adjusted for the set of covariates, study setting was not associated with a significant change in the hazard of death during the follow-up period (HR: 1.28 95% CI: 0.76, 2.13).

**Table 2:** Estimated Hazard Ratios Modeling Time to Death (with time-fixed study setting variable), Time to First Hospitalization (with time-fixed study setting variable), and Time to First Hospitalization (with time-varying study setting variable)

	Time-Fixed: Time to Death HR (95% CI)	Time-Fixed: Time to First Hospitalization HR (95% CI)	Time-Varying: Time to First Hospitalization HR (95% CI)
Study Setting (RCT vs. Cohort Study) Non-Time-Varying	1.28 (0.76, 2.13)	**0.72** **(0.58,0.89)**	N/A
Study Setting (RCT vs. Cohort Study) Time-Varying	N/A	N/A	**0.42** **(0.31, 0.58)**
Sex (Male vs. Female)	0.92 (0.59,1.45	**1.25** **(1.02,1.54)**	**1.25** **(1.02, 1.54)**
Site Type (Non-Urban vs. Urban)	0.79 (0.49,1.26)	1.04 (0.84,1.28)	1.05 (0.85, 1.30
SES Score (Increasing SES Status)	0.84 (0.70,1.01)	0.94 (0.86,1.02)	0.94 (0.87, 1.02)
Breastfeeding Strategy (Exclusively Formula Fed vs. Ever Breastfed)	1.19 (0.65,2.18)	**1.37** **(1.03,1.83)**	**1.34** **(1.01, 1.79)**
Pregnancy ARV Strategy (Reference: None)			
Pregnancy ARV Strategy (ZDV Only)	0.86 (0.25,2.98)	1.00 (0.56,1.77)	1.03 (0.58, 1.83)
Pregnancy ARV Strategy (3-drug ART)	0.60 (0.18,1.99)	0.76 (0.44,1.32)	0.82 (0.47, 1.41)
Birthweight Status (Not Low-Birthweight vs Low-Birthweight)	0.58 (0.34,0.97)	1.01 (0.76,1.33)	1.00 (0.76, 1.33)

### Morbidity

**Fig. 1:**
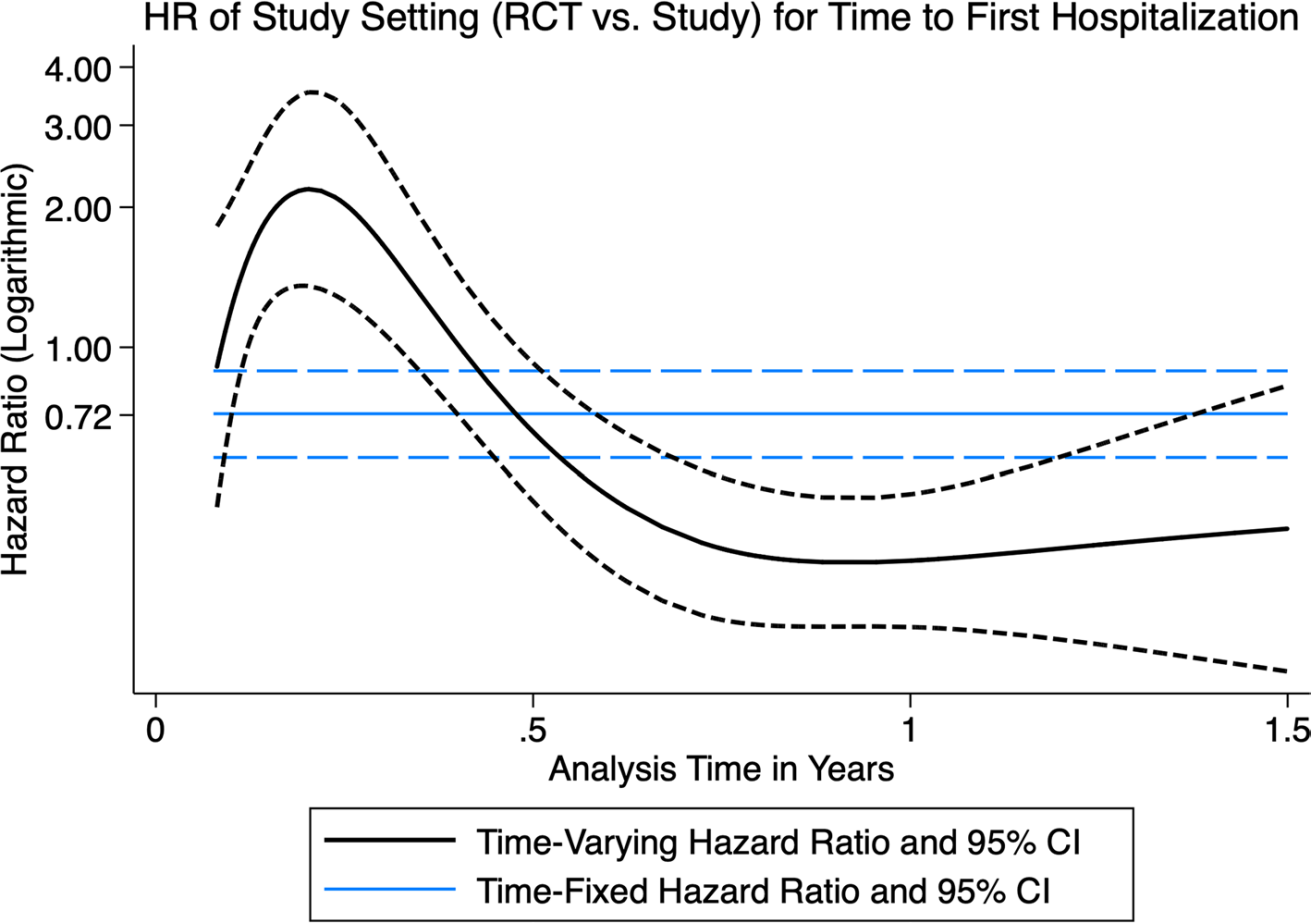
Restricted Cubic Spline Transformation of Hazard Ratio of Study Setting Over Analysis Time

In the Cox proportional hazards model fitted for time to first hospitalization, and adjusted for ARV strategy during pregnancy, sex, socioeconomic status, and infant feeding strategy, enrollment into the RCT was associated with a time-fixed hazard ratio of 0.72 (95% CI: 0.58, 0.89). An interaction term between analysis time in years and study setting was generated and fitted with the original covariates. The re-fitted model reported an an HR of 0.42 (95% CI: 0.31, 0.58) for the interaction term. A visual assessment of the change in HR over time suggested that the HR is initially greater than 1 during the first six months of enrollment and subsequently decreases well below the null (Fig. 1). Given that the RCT and the cohort study enrolled HEU infants at birth, this excess risk of hospitalization in the RCT setting occurs within the first six months of life. After this, the point estimate for the HR departs significantly from the null and remains below the null until the end of the analysis time.

## Treatment effects

### Mortality

The causal effect of the RCT on mortality was obtained through an inverse probability weighted estimator. Had everyone been enrolled in the cohort study, the average risk of death would have been 0.02 (95% CI: 0.01, 0.02). No difference in mortality was attributable to enrollment into the RCT (ATE: 0.00 (95% CI: -0.00, 0.01) (Table 3).

**Table 3:** Treatment effect analysis of morbidity and mortality

	**Coefficient**	**P >|z|**	**95% CI**
Risk of Mortality in the cohort study^a^	0.02	< 0.001	(0.01, 0.02)
Difference in Mortality caused by RCT^b^	0.00	0.263	(-0.00, 0.01)
Risk of Morbidity in the cohort study^a^	0.12	< 0.001	(0.10, 0.13)
Difference in Morbidity caused by RCT^b^	-0.03	0.001	(-0.06, -0.01)

^a^Indicates potential-outcome mean (POM) had all children been enrolled into the cohort study

^b^Indicates average treatment effect (ATE) attributable to enrollment into the RCT

### Morbidity

A similar approach was used to estimate the causal effect of enrollment into the RCT on the risk of hospitalization. Had everyone been enrolled in the cohort study, the average risk of hospitalization would have been 0.12 (95% CI: 0.10, 0.13). The causal risk difference for hospitalization attributable to enrollment into the RCT was -0.03 (95% CI: -0.06, -0.01), suggesting a decrease in the risk of hospitalization by approximately 29.77% (Table 3).

### Sensitivity analysis

When 43 infants who experienced outcomes within 30 days of enrollment were excluded, conclusions regarding the effect of study setting on mortality remain null while the effect of enrollment into the RCT on morbidity become even more pronounced. In this smaller population, the average treatment effect of the RCT on mortality was 0.00 (95% CI: -0.01, 0.01) while the average treatment effect on morbidity was -0.05 (95% CI: -0.07, -0.02), suggesting a decrease in the risk of hospitalization by approximately 39.45% (Table 4).

**Table 4:** Sensitivity analysis of treatment effects excluding infants experiencing outcomes within 30 days of enrollment

	**Coefficient**	**P >|z|**	**95% CI**
Risk of Mortality in the cohort study^a^	0.02	< 0.001	(0.01, 0.02)
Difference in Mortality caused by RCT^b^	0.00	0.947	(-0.01, 0.01)
Risk of Morbidity in the cohort study^a^	0.12	< 0.001	(0.10, 0.13)
Difference in Morbidity caused by RCT^b^	-0.05	< 0.001	(-0.07, -0.02)

^a^Indicates potential-outcome mean (POM) had all children been enrolled into the cohort study

^b^Indicates average treatment effect (ATE) attributable to enrollment into the RCT

## Discussion

Our study provides a quantitative estimate of how enrollment into the clinical trial setting affected mortality and morbidity compared to enrollment in an observational cohort study. We note in particular that the most pronounced difference between the RCT setting and the cohort study setting was the standard of care offered between the two. The RCT involved an extensive level of in-person follow up that drew on the resources of the trial to offer a level of care and follow-up that was markedly different than routinely accessed care in Botswana. Although mortality did not differ, hospitalization decreased between 30–40% among children participating in the RCT. Given that the cohort study was never intended to measure the effect of CTX as was the case for the RCT, it is impossible to determine whether these domains of the RCT influenced its effect estimate of CTX on mortality and morbidity in HEU infants. However, by isolating the effect of enrollment into the RCT setting, we provide evidence that motivates future assessment of these domains on the external validity of RCTs.

### Effects on mortality

There was no evidence of a protective effect on mortality from the clinical trial. Given the highly conservative nature of the exclusion criteria to ensure the validity of the inferences made, many infants who died in both settings were excluded and thus we had limited power to detect any meaningful differences in mortality between the trial and the study settings after these children were excluded.

### Effects on morbidity

Both the primary and sensitivity analyses of morbidity suggested a strongly protective effect from enrollment into the RCT. The estimate of the causal reduction in the risk of hospitalization (29.77% in the primary analysis and 39.45% in the sensitivity analysis) remained robust, as did the time-varying effects of the trial setting. It is unlikely that a high early HR associated with the RCT is driven by a biological mechanism given that children with a low-likelihood of survival were excluded from both studies. It is more likely that this early risk is driven by the intentional, physician-directed hospitalization of study subjects in the RCT, a phenomenon that was not part of the cohort study, by design. After this time period, the HR continued to remain below the null suggesting that the long-term and involved care provided by the RCT protected its subjects from excess hospitalization between six and eighteen months of life. The most plausible interpretation of our results, therefore, is that physician availability in the RCT allowed better identification of at-risk children, and in some cases led to early referral of those who were acutely or chronically ill, had missed vaccinations, or were failing to meet growth or developmental standards for age. This degree of management and care was ultimately associated with a lower overall risk of hospitalization through 18 months. This phenomenon, which reflects an outcome that could be achieved in health care settings when standards of care are uniformly and consistently applied, highlights the need for implementation science research to identify barriers to implementation of standards of care in real-world health care settings.

### Strengths and limitations

Strengths of our study included the use of two existing cohorts in comparable locations in Botswana that were followed during an overlapping calendar period and captured similar health outcomes, allowing us to largely isolate the difference between in-person (RCT) and telephone (cohort study) follow-up. Both had additional favorable characteristics, including the fact that the RCT showed no difference between CTX vs. placebo (allowing us to use data from both arms), and the fact that the cohort study’s telephone follow-up was deliberately intended to have no impact on clinical care delivery in its design and implementation. Both had extremely high rates of follow-up and completeness of data, which allowed meaningful assessment of morbidity and mortality through 18 months. Only 5% of children were lost to follow up in the RCT and vital statuses at 24 months were missing for only 0.5% of children in the cohort study.

The results of this study are specific to a high HIV burden, low-resource context. The protective effects of the trial setting here are relative to routine care in Botswana. These differences may not be observed in high-income settings, where the difference in care offered in a clinical trial may not differ appreciably from care offered in the routine delivery of health care in a non-study setting. Additional limitations include the different study sites used for each trial, which could have led to several types of bias if there were differences in care or disease risk by site. However, the sites for both studies were similar in size and in resource availability and we are not aware of differences in health metrics that would have been likely. Aside from these, there is also unobserved confounding which may have increased the likelihood of assignment to the trial or the study setting.

There were also some baseline differences between trial participants. These included slightly higher access to ART in pregnancy among the mothers of enrolled children in the RCT, which could have had some unmeasured or residual confounding of health outcomes for children later in life [13]. Mothers in the RCT were of a slightly higher socioeconomic status than mothers in the cohort study, but an average difference of less than 0.2 points on a 7-point SES scale is unlikely to suggest a substantive difference in SES between the RCT and cohort study populations. To partially alleviate the concern of overrepresentation of healthier children in the RCT, infants from both studies who died within 30 days of birth were excluded from the analysis. Finally, the results of the sensitivity analysis conducted suggested that the protective effects of trial care may occur further into enrollment, and in the case of the study population, further into early life.

### Future directions

Our work provides motivation for a number of future research questions related to this topic. First, can our finding that enrollment into an RCT provides a morbidity benefit compared to accessing routine care be replicated in other low-resource contexts, where the discrepancy is more likely to be pronounced? Previous studies have characterized this association in high-income settings through matched-historical cohort studies or pooling participants of multiple clinical trials, but these methods are limited by the retrospective matching and the heterogeneity introduced from the conduct of different trials [14–16]. There is also a need for future research to assess differences between the populations enrolled into the trial setting that extend beyond the eligibility criteria reported to ensure further overlap between study and non-study populations. Finally, this research motivates the investigation of whether these domains of difference impact the effect estimates of interventions obtained in RCTs. For example, did CTX demonstrate no measurable effect in the RCT because any effect it might have had was masked by the improved standard of care? Alternatively, would CTX have had an effect in the routine care setting, protecting HEU infants from longer-term morbidity that would have otherwise been identified, prevented, or treated early in the RCT setting? While we are unable to address these questions with the data available, we believe that their answering might further motivate the use of pragmatic trials, emulated RCTs and real-world evidence platforms, and implementation science to ensure that the results of RCTs are valid outside of their immediate context.

## Conclusions

Our results provide empirical evidence to help quantify the differences in morbidity and mortality rates between clinical trials and routinely accessed care in resource constrained settings. For children who are HEU, we estimate that there was nearly a 30% reduction in hospitalization by participating in a clinical trial. We demonstrate that the morbidity rate of HEU infants obtained in the RCT setting was not externally valid for the relevant, non-trial population. While we are unable to determine whether the effect estimate of the intervention was externally valid, we propose that this phenomenon can occur if the benefits of the enhanced standard of care could independently affect relevant outcomes and thus mask the effect of the intervention in routine care settings. However, more work is needed to determine if this is plausible. Outcomes in the non-trial setting may fall short of what is achievable for a myriad of reasons, including limited health personnel, inadequate training of staff, supply chain shortages, or barriers to accessing care. While pragmatic trials are warranted to ensure external validity of study findings, it is equally important to conduct implementation science research when results of clinical trials are not routinely observed in real-world settings.

## Data Availability

The datasets used and/or analyzed during the current study are available from the corresponding author on reasonable request.
